# Drivers’ Evaluation of Different Automated Driving Styles: Is It Both Comfortable and Natural?

**DOI:** 10.1177/00187208221113448

**Published:** 2022-07-11

**Authors:** Chen Peng, Natasha Merat, Richard Romano, Foroogh Hajiseyedjavadi, Evangelos Paschalidis, Chongfeng Wei, Vishnu Radhakrishnan, Albert Solernou, Deborah Forster, Erwin Boer

**Affiliations:** 414566 University of Leeds, UK; 414566 University of Leeds, UK; 414566 University of Leeds, UK; 414566 University of Leeds, UK; Birmingham City University, UK; 414566 University of Leeds, UK; 414566 University of Leeds, UK; Queen’s University Belfast, UK; 414566 University of Leeds, UK; 414566 University of Leeds, UK; Entropy Control, Inc., San Francisco, California, USA; Entropy Control, Inc., San Francisco, California, USA

**Keywords:** highly automated driving, driving style, comfort, naturalness, sensation seeking

## Abstract

**Objective:**

This study investigated users’ subjective evaluation of three highly automated driving styles, in terms of comfort and naturalness, when negotiating a UK road in a high-fidelity, motion-based, driving simulator.

**Background:**

Comfort and naturalness play an important role in contributing to users’ acceptance and trust of automated vehicles (AVs), although not much is understood about the types of driving style which are considered comfortable or natural.

**Method:**

A driving simulator study, simulating roads with different road geometries and speed limits, was conducted. Twenty-four participants experienced three highly automated driving styles, two of which were recordings from human drivers, and the other was based on a machine learning (ML) algorithm, termed Defensive, Aggressive, and Turner, respectively. Participants evaluated comfort or naturalness of each driving style, for each road segment, and completed a Sensation Seeking questionnaire, which assessed their risk-taking propensity.

**Results:**

Participants regarded both human-like driving styles as more comfortable and natural, compared with the less human-like, ML-based, driving controller. Particularly, between the two human-like controllers, the Defensive style was considered more comfortable, especially for the more challenging road environments. Differences in preference for controller by driver trait were also observed, with the Aggressive driving style evaluated as more natural by the high sensation seekers.

**Conclusion:**

Participants were able to distinguish between human- and machine-like AV controllers. A range of psychological concepts must be considered for the subjective evaluation of controllers.

**Application:**

Insights into how different driver groups evaluate automated vehicle controllers are important in designing more acceptable systems.

## INTRODUCTION

With higher SAE level AVs (SAE International, 2016), drivers will inevitably lose the controllability of the vehicle, and the role of human drivers will shift from active controllers of the vehicle, towards passive observers and passengers (Elbanhawi et al., 2015; Kaber & Endsley, 2004). There are several subsequent concerns that might hinder the deployment of these vehicles, such as users’ experience of comfort inside the AV (Elbanhawi et al., 2015). Comfort is crucial for an AV’s implementation, as it is found to be correlated with trust and acceptance (Paddeu et al., 2020; Siebert et al., 2013), important elements for encouraging public uptake of these new forms of mobility (Madigan et al., 2016).

Although there is currently no commonly agreed definition for comfort in this context, some suggestions exist. Under the context of automated driving, Hartwich et al. (2018) summarised driving comfort as ‘*a subjective, pleasant state of relaxation given by confidence and an apparently safe vehicle operation, which is achieved by the removal or absence of uneasiness and distress’* (p. 1019).

For automated vehicles, however, comfort is not simply limited to physical aspects of the vehicle, such as good seat design (Ebe & Griffin, 2001), or acceptable levels of engine noise, and vehicle vibrations (Qatu, 2012). These features are mentioned in studies of traditional, manually operated, road vehicles and also in other domains, for example, cabin noise in aircraft (Pennig et al., 2012). Since the vehicle is no longer controlled by a human, it is important that its ‘driving behaviour’, and how it negotiates different road geometries, and traffic conditions, is considered pleasant, and rated positively by the user, ensuring it feels comfortable and safe (Elbanhawi et al., 2015; Summala, 2007). Other, more psychological, terms and concepts used in this context include ensuring the AV is considered reliable, and familiar, avoiding any sudden surprise behaviours, which are shown to enhance the acceptance, satisfaction and perceived safety of AVs (Carsten & Martens, 2018; Ramm et al., 2014).

One, relatively unexplored, concept in this context is ‘naturalness’ of the AV’s driving behaviour, which has been linked to the familiarity of the AV’s manoeuvres, for the user. Here, the familiarity of AV movements, rendered by mimicking human-like vehicle controls, is expected to fulfil human users’ anticipation of an AV’s behaviours, and result in positive subjective feedback (Butakov & Ioannou, 2015; Hartwich et al., 2018). Moreover, Elbanhawi et al. (2015) suggest that naturalness of automated driving is an important determinant of comfort. However, some empirical studies have shown that familiar automated driving manoeuvres do not always lead to higher subjective comfort (Hartwich et al., 2018), which suggests that more knowledge is needed on the link between these two concepts, since they will likely contribute to acceptance of future AVs.

From a technical perspective, there are a large number of automated driving styles that could be generated for such investigations. Taking motion planning as an example, the generated driving behaviour of AVs could be robotic, with algorithm-optimised trajectories, based solely on sensory information provided by lasers, radars and cameras, to adapt to the environment (e.g., Urmson et al., 2008). Alternatively, these may mimic a human driver’s average behavioural patterns, by training models, based on real human driving data (e.g., Hajiseyedjavadi et al., 2021; Rehder et al., 2017; Wei et al., 2019). Personalisation of driving styles can also be achieved by using users’ own driving style in the model development loop (e.g., Menner et al., 2019).

Studies on manual driving suggest that participants’ reported levels of comfort are also linked to the vehicle’s ‘driving style’ (Bellem et al., 2018), which is defined as the driving habits of the driver, such as their preferred speed, threshold for overtaking, headway distance and tendency to violate traffic regulations (Elander et al., 1993). In highly automated vehicles, the use of such driving styles has been reported to enhance driving comfort of passive users (Bellem et al., 2018).

Research has revealed the existence of several driving styles, associated with different character traits of human drivers, loosely linked to defensive (less sudden acceleration and deceleration) and aggressive (higher acceleration and more sudden braking) driving behaviours (Murphey et al., 2009). Results also suggest that different automated driving styles are sometimes found to be preferred by different groups of users, when evaluated in terms of comfort, safety and pleasantness, although findings are inconclusive. For example, a more defensive driving style, with slower lane changing features, and lower acceleration, was favoured by most participants, when compared with a higher acceleration, more assertive, driving style (Rossner & Bullinger, 2020). Moreover, Hartwich et al. (2018) found that familiar driving styles (a replay of participants’ own driving) were more favoured by younger drivers (25–35 years), while faster and unfamiliar automated driving styles (that of the younger drivers) were preferred by older drivers (65–84 years). Therefore, Hartwich et al. (2018) suggest that solely mimicking drivers’ personal manual driving habits may not be suitable for all age groups. Using a more comprehensive set of vehicle kinematics, Bellem et al. (2018) manipulated the initiation time and strength of acceleration and jerk of three manoeuvres on the highway (i.e., lane changes, accelerations and decelerations). These authors recommend a number of configurations for comfortable driving experiences, such as minimising jerk for acceleration and deceleration manoeuvres, lowering acceleration and providing action feedback, which is when maximum acceleration is applied at the early stages of a lane change manoeuvre.

As outlined above, most of the existing studies considering users’ responses to different driving styles of AVs have compared different replays of drivers’ manual driving performance. To date, there has been little comparison of user preferences for machine- versus human-like AV-driving styles. An important consideration here is the balance between what is expected from users about the acceptable driving style of an AV, compared to that of a human driver. For example, studies have shown that an AV controller that precisely follows the lane centre, is considered more competent, compared to those with less accurate lane-tracking and more lateral drifts from the centre lane (Price et al., 2016). Therefore, from a human factors perspective, more research is warranted to understand what types of driving styles and behaviours of machine- and human-like driving are considered more comfortable and natural, and whether these are linked to the particular driving environment being negotiated by the AV.

Users’ perception of an AV’s driving style is known to be influenced by both objective and subjective factors. For example, road furniture and geometry are known to influence ratings of safety and comfort (Hajiseyedjavadi et al., 2021) and physiological response (Beggiato et al., 2019; Radhakrishnan et al., 2020), while a number of studies have shown a correlation between personality traits such as Sensation Seeking (Arnett, 1994) and preferred driving style. For example, in manual driving; drivers with high sensation seeking scores are found to drive in a riskier and more aggressive manner and at higher speeds, while low sensation seekers have a tendency to drive more slowly (Louw et al., 2019; Taubman-Ben-Ari et al., 2004; Zuckerman & Neeb, 1980). However, results are mixed regarding preferences for AV-driving styles. For example, Yusof et al. (2016) reported that both assertive and defensive drivers, characterised by higher and lower sensation seeking scores, respectively, showed a consistent preference for a defensive (and not assertive) AV-driving style. Therefore, in addition to considering user response to two human-like and one machine-like AV controller, this study assessed the effect of road geometries and users’ sensation seeking scores on such evaluations.

### Current Study

This study is based on data collected from a driving simulator study within the UK-funded HumanDrive project (TS/P012035/1). The main purpose of which was to develop, and evaluate, advanced AV controllers, imitating natural, human-like, driving styles. Two representative human-like driving styles were recorded, and replayed to participants. Response to these was compared to a machine-like, machine learning (ML)-built, driving style (Solernou et al., 2020).

The following research questions were addressed in the present study:

1. Are the three driving styles rated differently in terms of perceived comfort and naturalness?
2. Do environmental settings influence the comfort and naturalness of the three driving styles?
3. Do users’ sensation seeking propensities affect their ratings of comfort and naturalness towards the three driving styles?
4. Is a natural driving style also a comfortable driving style?

## METHODS

### Participants

Twenty-four participants (12 male, 12 female) aged between 20 and 49 years (M = 35.7, *SD* = 7.1) were recruited. All participants held a valid UK driving licence, with experience ranging from 2 to 27 years (M = 14.7, *SD* = 7.8). Reported annual driving distance ranged from 500 to 18000 miles (M = 7554.2, *SD* = 3982.7).

All participants were recruited by using the University of Leeds Driving Simulator database, and all provided informed consent to take part in the study. Each participant was compensated £30 for taking part in the study. This study was approved by the University of Leeds Ethics Committee (LTTRAN-086).

### Apparatus

The high-fidelity, motion-based University of Leeds Driving Simulator (UoLDS) was used in the experiment. The simulator’s vehicle cab is based around a 2006 Jaguar S-type, housed within a 4 m diameter, spherical projection dome. There are eight visual channels rendered at 60 frames/s, predominantly at a resolution of 1920 × 1200, providing a horizontal forward field of view of 270°. The simulator also incorporates an eight degree-of-freedom electrical motion system. The generated range of acceleration of the motion system is ±5.0 m/s^2^ (Jamson et al., 2007)

### Experimental Design

A fully within-participant experimental design was used in this study to investigate participants’ subjective evaluation of three different automated vehicle driving styles, described below. Participants were asked to use an eleven-point, Likert-type, scale, to rate how ‘comfortable’ and ‘natural’ each automated drive felt, as it negotiated the same stretch of road, in six separate drives, completed over 2 days.

#### Driving Styles

**Figure 1.**
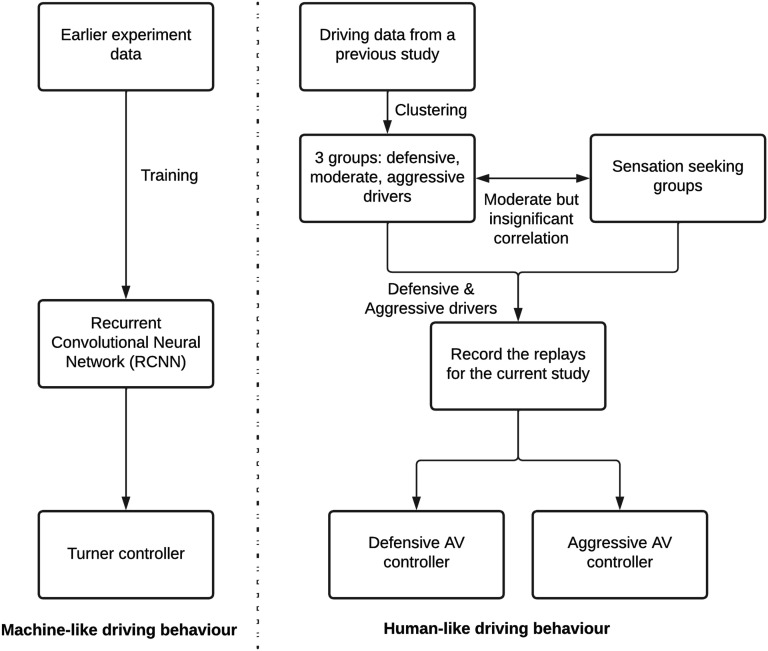
Overview of the development of the three AV controllers.

A machine learning (ML) based controller, and two human-driven controllers were developed for evaluation in this study. These controllers are described further below, and a diagram presenting the development procedure is shown in Figure 1.

##### The ML-Built Controller (Turner)

The ML controller was calibrated using a Recurrent Convolutional Neural Network (RCNN) that was capable of imitating the human driving behaviour, in terms of future yaw rate and speed demands. The RCNN was trained from data of 10 participants, from an earlier experiment of the project (see Solernou et al., 2020). This controller will be called the Turner controller from here on.

##### The Human-Like Controllers (Termed Aggressive and Defensive controllers)

The two human-like controllers were recorded drives of human participants, collected before the main study took place, which were then replayed to participants of this study. Previous studies have shown a positive correlation between speed choice and sensation seeking (Louw et al., 2019) as well as risk-taking behaviour in manual driving (Ge et al., 2014; Oppenheim et al., 2016; Riendeau et al., 2018; Ulleberg & Rundmo, 2003). To ensure that distinct differences in driving behaviour would arise between the two human-driven controllers, recruitment of participants used for the human-driven controllers was based on their sensation seeking scores.

Before recruiting participants for these replay drives, data from a previous study of the project was used to create clusters of driving behaviour (see Appendix A). These participants were clustered into three main groups: defensive, moderate and aggressive drivers. There was a moderate, but insignificant, correlation between participants’ sensation seeking scores, and cluster membership (r(14) = .429, *p < .*143). For example, we found that the aggressive driving cluster contained participants with higher sensation seeking scores. The absence of a significant correlation was likely due to the small sample size used in this study. Following this analysis, participants with higher sensation seeking scores from the aggressive cluster, and lower sensation seeking scores from the defensive cluster were contacted to participate in the replay recordings of the current study. In total, eight participants were recruited, four for each sensation seeking group (Table 1).

**TABLE 1:** Descriptive Statistics of the Participants Used for the Replay Recording Phase

	Gender		Age		AISS Score
	Male	Female	Mean	Std.	Mean
High sensation seeking	4	0	36.25	9.78	55.75
Low sensation seeking	2	2	52	6.73	45

*Note*. AISS scores were calculated based on drivers’ responses to Arnett Inventory of Sensation Seeking (AISS; Arnett, 1994), and scores were the sum of all responses to in total of 20 questions, with a higher score means higher sensation seeking propensities.

During the recording process, each participant drove the experimental route three times. The process took approximately 1 hour. After the data collection, the clustering process was applied again for the new data, to confirm the obtained driving behaviours belonged to the previously identified defensive and aggressive driver groups, respectively. Out of the eight participants recorded, the manual driving data of two participants (one per sensation seeking group, with scores of 59 and 43, respectively) which was closest to the median of the defensive and aggressive clusters, were selected as the representative driving styles for our two human-like controllers. It is worth highlighting that the selected drives were also checked to ensure that no unusual or unexpected manoeuvres existed along the drive. For the rest of this paper, the higher sensation seekers’ driving style will be termed Aggressive, and the lower sensation seekers’ driving style will be called the Defensive driving style.

#### Road Environment and Scenarios

**Figure 2.**
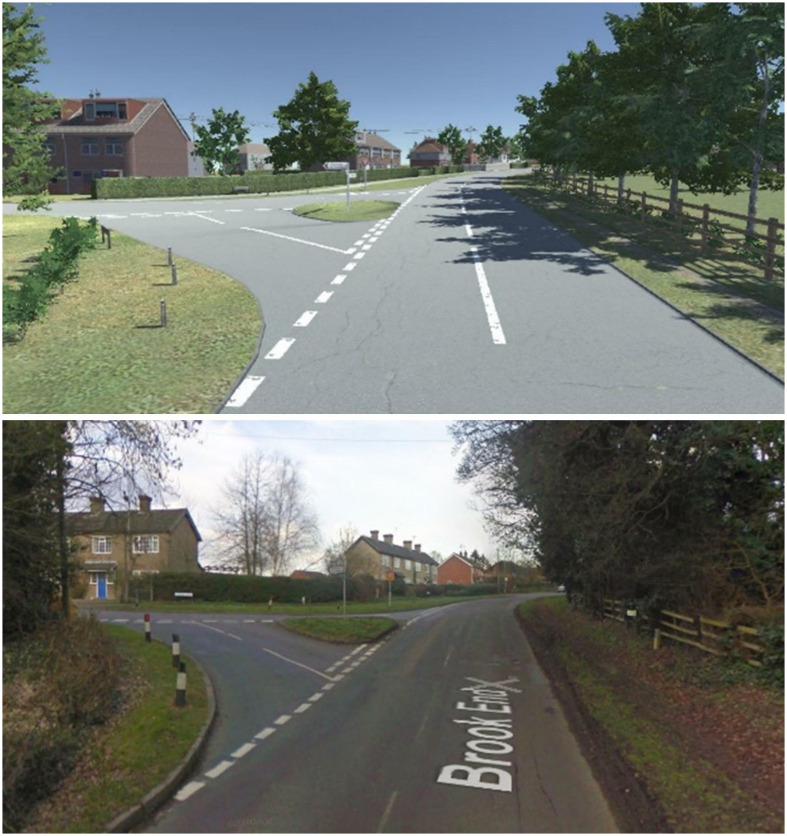
Example of the simulated (top) and real (bottom) road environments.

The simulated driving scene was modelled from real stretches of road around North Bedfordshire in the UK (Figure 2). Two loops, going North and South, were simulated, creating a virtual environment covering around 12 miles of driving. In the present work, however, only the North loop was included for the simulated drive, since it included the range of scenarios required for studying driver behaviour in response to changes in speed and geometry, and shortened the overall drive. This section of road was approximately 5 miles long, taking about 15 minutes to complete.

To understand user preferences for, and in response to, a wide range of road geometries and speed profiles, the layout of the North loop contained a combination of high-speed (60 mph) rural sections, with varying road curvature and more built-up, village sections, at a speed limit of 40 mph (Table 2).

**TABLE 2:** The Speed Limit and Geometrical Details of the Simulated Road

Zone	Curve Radius	Curve Direction	Road Type	Speed Limit (mph)	Road Context
1	300–800 m	Left	Rural	60	Kerb + grass and the bridge in the middle of the area
2	Straight	Straight	Rural	60	Kerb + grass with hedge far from the road edge
3	<150 m	Left	Rural	60	Kerb + grass and trees far from the road edge
4	<150 m	Left	Rural	60	Kerb + grass with hedge quite far from the road edge
5	300–800 m	Right	Rural	60	Kerb + grass with hedge quite far from the road edge
6	<150 m	Right	Village	40	Kerb + grass and some structures far from the road edge
7	<150 m	Left	Village	40	Kerb + grass
8	200–300 m	Right	Village	40	Kerb + grass and hedge around 1–2 m from the road edge
9	Straight	Straight	Village	40	Kerb + grass and fence quite close to the road edge
10	150–200 m	Right	Village	30	Kerb + pavement and village structures far from the road edge
11	150–200 m	Right	Village	30	Parked cars zone
12	300–800 m	Right	Village	30	Kerb + grass and village structures
13	200–300 m	Left	Rural	40	Grass, bushes and trees not close to the road edge
14	300–800 m	Right	Rural	40	Grass and hedge far from the road edge
15	<150 m	Right	Rural	60	Grass and trees far from the road edge
16	<150 m	Left	Rural	60	Hedge at the road edge
17	150–200 m	Right	Rural	60	Grass and hedge far from the road edge
18	300–800m	Left	Rural	60	Grass and bushes around 2 m from the road edge
19	Straight	Straight	Rural	60	Fence around 1–2 m from the road edge
20	Straight	Straight	Rural	60	Hedge at the road edge and an intersection at the end of the section
21	N/A	N/A	University	30	Mini roundabout and road markings
22	<150 m	Left	University	30	Parked cars zone
23	300–800 m	Left	University	30	Kerb + pavement
24	Straight	Straight	University	30	Kerb + pavement

### Variables

The dependent variables were comfort and naturalness of the driving experience, for each controller. A search of the literature at the time of study design revealed an absence of a formal, and universally agreed, description for the two terms. To ensure that the same term was understood by all participants, we therefore used a small expert group within the project team to define the two terms, and included this information in the participant briefing sheet:

i. Comfortable driving was defined as ‘a driving style that does not cause any feeling of uneasiness or discomfort’;
ii. Natural driving was defined as ‘a driving style that is closest to your own driving’.

**Figure 3.**
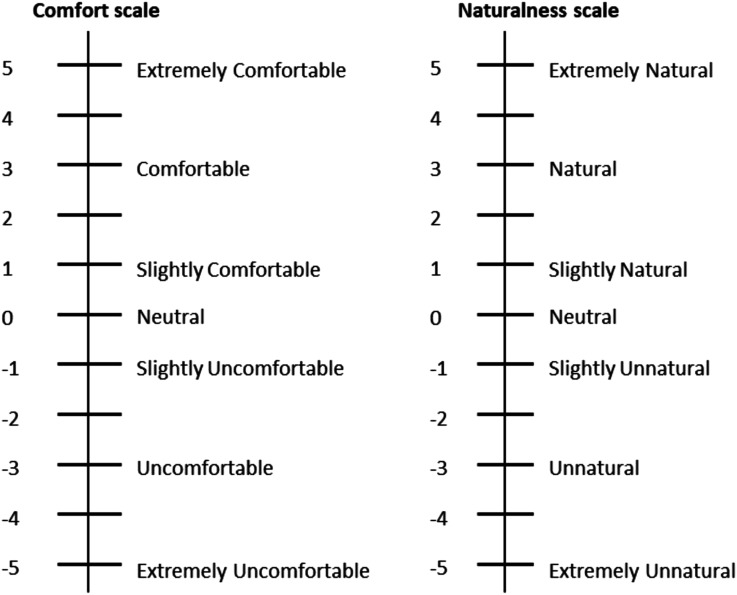
The comfort and naturalness scales used in the study.

Participants evaluated each controller, in two ways: (i) after each drive, participants were asked to provide an overall rating, based on their entire driving experience and (ii) throughout the drive, immediately after they heard a short auditory beep, which was played via the car’s speakers, corresponding to 24 relevant sections in the drive (Table 2). They were taught to use a Likert-type scale for guiding their responses, providing a number between −5 (Extremely Uncomfortable/Unnatural) and +5 (Extremely Comfortable/Natural) (Figure 3).

Participants also completed the Arnett Inventory of Sensation Seeking questionnaire (Arnett, 1994) after they finished the last drive. This questionnaire includes twenty items, and four response options for each item, ranging from 1 (does not describe me at all) to 4 (describes me very well). Reverse-worded items were further reverse-coded. We used the sum score of these items to characterise sensation seeking tendency, with a higher score indicating a higher sensation seeking tendency.

### Procedure

**Figure 4.**
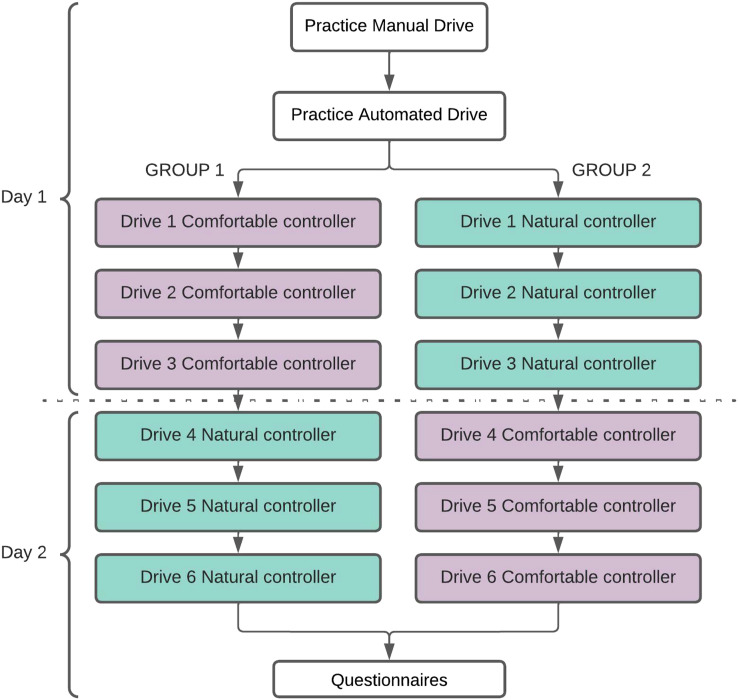
The overall experimental procedure, including the order of drives.

To reduce the effect of fatigue on participants, the study was conducted over two separate days (M = 6.75 days apart, *SD* = 2.17), with data collection lasting about 1.5 hours on each day. Participants evaluated the three driving styles twice: once in terms of comfort, and once in terms of naturalness, with half of the participants evaluating in terms of comfort on day 1, and the other half on day 2, (Figure 4).

Upon arrival on the first day, each participant received a written and verbal briefing of the study, including how to use the subjective scale, and provided their written consent to take part in the experiment. They then started the simulator experiment with a practice drive in manual driving, followed by a practice ride in an automated driving mode. A researcher accompanied participants during the practice session, which lasted 20–30 minutes. Following the practice drive, the researcher left the simulator dome, and the participant started the first of three experimental drives, one for each controller. The order of the three automated driving styles was counterbalanced across participants, and participants left the simulator dome after each drive, to reduce fatigue effects. After the second day’s experiment, participants were asked to complete a set of questionnaires, including the sensation seeking questionnaire. The data from the other questionnaires is not reported here.

## RESULTS

The main aim of the analyses was to assess users’ evaluation of the three automated controllers, in terms of comfort and naturalness. Participants’ subjective feedback about the driving styles adopted by the controllers was provided in two ways: (i) an overall evaluation of the controller, after finishing the entire drive and (ii) 24 responses, based on the 24 auditory beeps throughout each drive, which prompted a response for each of the different driving zones. Two statistical tests were used: the Wilcoxon signed-rank test was used for the overall evaluation provided at the end of each drive, and the Generalised Estimating Equation (GEE) (Liang & Zeger, 1986) was used for the 24 individual evaluations provided during the drive (see configurations of GEE in Appendix B).

### Subjective Evaluations of the Driving Styles

**Figure 5.**
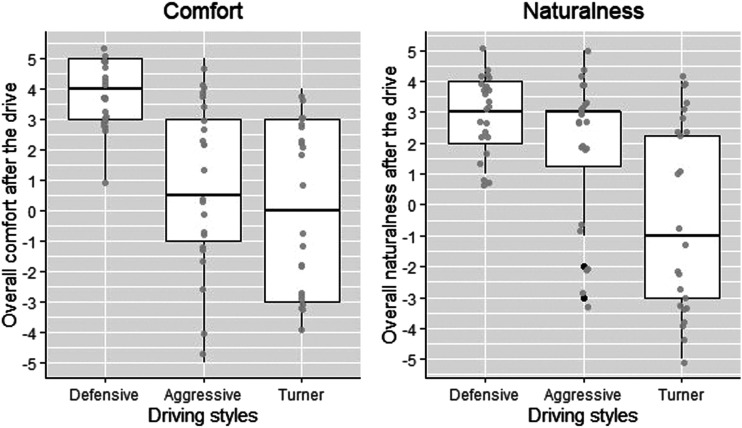
Evaluation of each controllers’ driving style, in terms of its overall comfort (left) and overall naturalness (right). Horizontal lines inside each box represent the median values. Whiskers denote a distance of 1.5 times interquartile range (IQR) above the upper quantile up to the largest observation, or below the lower quartiles up to the smallest value. Grey dots represent data points (with small variations added to the position to avoid overlapping), while black points represent outliers.

Table 3 shows results of the Wilcoxon signed-ranks test on matched-pairs, and Figure 5 shows box plots of overall comfort and naturalness evaluation of the three driving styles.

**TABLE 3:** Wilcoxon Signed-Rank Test Results for Overall Comfort and Naturalness

Driving Style	z	p	r
Comfort (overall)			
Defensive vs Turner	4.27	0.000*	0.87
Defensive vs Aggressive	4.11	0.000*	0.84
Aggressive vs Turner	0.70	0.490	0.14
Naturalness (overall)			
Defensive vs Turner	3.67	0.001*	0.75
Aggressive vs Turner	2.44	0.010*	0.50
Defensive vs Aggressive	2.25	0.020*	0.46

*Note*. * *p < .*05. Orders of paired comparison are based on z values.

Regarding overall comfort, the Wilcoxon test showed significantly higher evaluation for the Defensive controller, compared to the Aggressive and the Turner controllers (both *p < .*001). There was no significant difference between the Aggressive and Turner controllers (*p < .*49). Regarding overall naturalness, the Wilcoxon test showed a significantly higher evaluation for the Defensive controller, compared to the Turner (*p < .*001), and a higher evaluation than the Aggressive controller (*p < .*02). A significantly lower score for the Turner than the Aggressive controller (*p < .*01) was also revealed.

Figure 5 shows that the evaluation of the Defensive driving style was relatively consistent across participants. By contrast, the evaluation for the Aggressive and Turner controllers was more variable, with a bimodal pattern observed in response to naturalness of the Aggressive, and the comfort and naturalness of the Turner. To understand this further, we conducted additional analyses by taking participants’ personality trait into account.

Table 4 presents the GEE results for the repeated ratings of comfort and naturalness. Regarding comfort, the probability of reporting high levels of comfort was significantly higher for the Defensive controller, compared to both the Turner (OR = 7.21, *p* < .001) and Aggressive controllers (OR = 4.01, *p* < .001). Comfort ratings were also slightly higher for the Aggressive, than the Turner, controller (OR = 1.80, *p* < .04). Regarding naturalness, both the Defensive and Aggressive controllers were more likely to be rated more natural, when compared to the Turner controller (OR = 4.98, *p* < .001; OR = 2.59, *p* < .002). The Defensive controller also had a higher probability of being assessed as more natural than the Aggressive driving style (OR = 1.92, *p* < .01).

**TABLE 4:** GEE Model Parameter Estimates and Odds Ratios for Repeatedly Reported Comfort and Naturalness

Driving Style	Coefficient	SE	Wald	Sig	Odds Ratio (OR)
Comfort					
Defensive vs Turner	1.975	0.238	68.847	0.000*	7.206
Defensive vs Aggressive	1.388	0.234	35.111	0.000*	4.007
Aggressive vs Turner	0.587	0.285	4.258	0.039*	1.799
Naturalness					
Defensive vs Turner	1.606	0.232	48.113	0.000*	4.980
Aggressive vs Turner	0.953	0.305	9.745	0.002*	2.593
Defensive vs Aggressive	0.653	0.255	6.560	0.010*	1.921

*Note*. *p < .05. Orders of paired comparison are based on the odds ratios.

**Figure 6.**
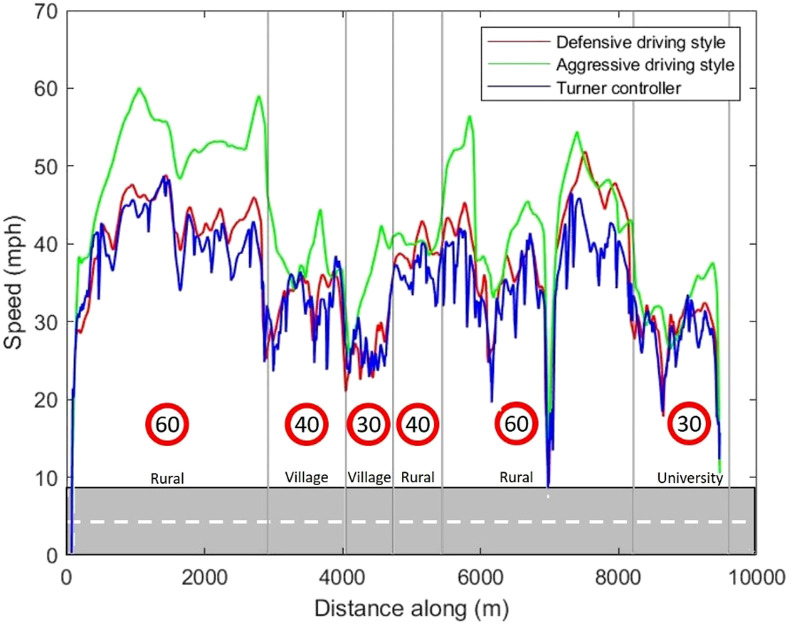
The speed profiles of the controllers.

**Figure 7.**
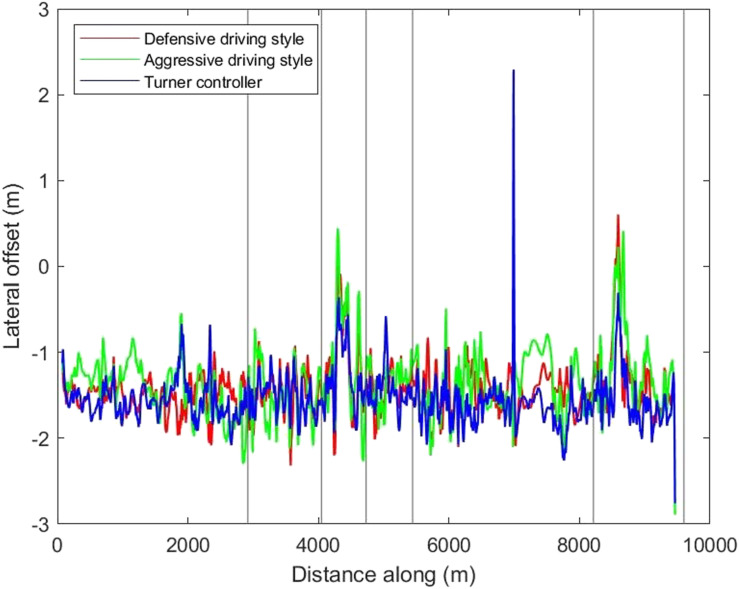
The lateral offset profiles of the controllers.

To further understand whether subjective evaluation was due to any differences in the driving styles of the controllers, the vehicle kinematics, including the speed and lateral offsets of all three controllers, were inspected (Figures 6 and 7). The interpretations provided in this section were based on visual observations of the controllers’ kinematic characteristics only, and no formal analyses were conducted. Figure 6 shows that, overall, speed was higher in the Aggressive driving style, compared to the other two controllers. The Defensive and Turner controllers had similar increasing or decreasing trends in speed, for the same road sections, with smoother patterns (i.e., less frequent and gentler fluctuations in speed) seen for the Defensive controller. There was not much difference in the observed lateral offset of the three controllers (Figure 7).

### The Effect of Road Environment on Subjective Evaluation

As shown in Table 2, the simulated road included a range of road environments divided into five main categories: (i) road type, (ii) speed limit, (iii) road context, (iv) curve direction and (v) curve radius. For simplicity, only two categories were included in this analysis, as follows:

i. road type (rural and village), which differed by posted speed limit (60 mph vs 40 mph) and roadside furniture (see Table 2);
ii. curve radius (five levels, varying from straight sections to curves of less than 150 m).

Road type was included as the representative of speed limit and road context, supported by the strong (r = 0.88, *p* < .001) and medium (r = −0.05, *p* < .001) correlation between road type and the two categories (speed limit, road context), respectively. The University road type was excluded from analysis, due to the small number of sections falling into this category. The direction of a curve was also not included as a factor, as it was not expected to have a significant influence on results. It is worth mentioning that the number of road sections in each level outlined above was not equal, since the road was a replication of the real world.

**Figure 8.**
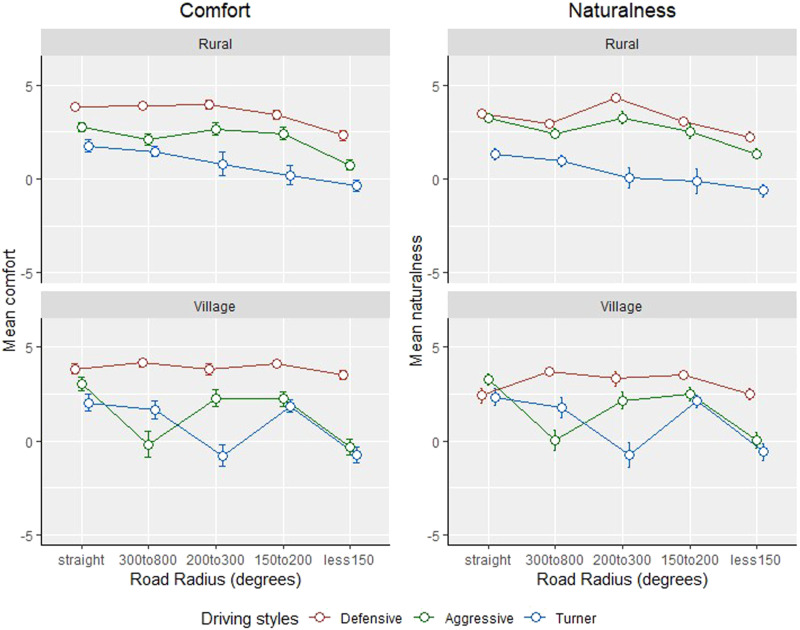
Mean evaluation scores for comfort (left) and naturalness (right) for each driving style, for the different road environments. Error bars indicate standard error of the data.

Figure 8 shows the average comfort and naturalness ratings for the three driving styles, for the different road sections. An overall pattern was observed, such that, with increasing curve radius, there was a mild reduction in both comfort and naturalness ratings for all controllers, especially in the Rural areas. This pattern was not apparent in the Village areas, apart from two unexpected fluctuations. Inspection of the vehicle-based metrics showed a high speed for the Aggressive controller in the 200–300 Curve Radius section, and a suddenly changing speed of the Turner in the 300–800 Curve Radius section (for further evaluation of these, see Appendix C).

Table 5 shows the results of the GEE models, which showed that the effect of driving style, on comfort and naturalness ratings, was significant for both the Rural and Village road sections (all *p* < .001). In the Rural sections, which had a generally higher speed limit than the Village sections, there was less difference in odds ratios between the Aggressive and the Turner controllers for the gentler roads (i.e., Straight and Curve Radius 300–800), but this difference was more prominent for the shaper road sections (i.e., Curve Radius 150–200, and 200–300). It is also worth highlighting the preference for the Defensive controller over the Turner, where the odds ratios are seen to be larger with increasing road curvatures. However, these differences in controllers were not observed for the sharpest Rural section (i.e., less than 150).

**TABLE 5:** The GEE Model Parameter Estimates and Odds Ratios, for Comfort and Naturalness in Rural and Village Roads with Different Curvatures

		Comfort						Naturalness					
Environment	Curvature	N	Driving Style	Coefficient	SE	Sig	OR	N	Driving Styles	Coefficient	SE	Sig	OR
Rural	Straight	216	Defensive vs Turner	1.662	0.326	0.000*	5.270		Defensive vs Turner	1.709	0.347	0.000*	5.522
			Defensive vs Aggressive	0.969	0.293	0.001*	2.636	215^a^	Aggressive vs Turner	1.569	0.365	0.000*	4.801
			Aggressive vs Turner	0.693	0.366	0.058	1.999		Defensive vs Aggressive	0.140	0.253	0.580	1.150
	300 to 800	288	Defensive vs Turner	1.926	0.289	0.000*	6.860		Defensive vs Turner	1.316	0.307	0.000*	3.727
			Defensive vs Aggressive	1.350	0.290	0.000*	3.856	288	Aggressive vs Turner	1.141	0.342	0.001*	3.129
			Aggressive vs Turner	0.576	0.310	0.063	1.779		Defensive vs Aggressive	0.175	0.301	0.561	1.191
	200 to 300	72	Defensive vs Turner	2.812	0.483	0.000*	16.650		Defensive vs Turner	4.067	0.624	0.000*	58.356
			Aggressive vs Turner	1.422	0.506	0.005*	4.146	72	Aggressive vs Turner	2.441	0.667	0.000*	11.486
			Defensive vs Aggressive	1.390	0.411	0.001*	4.016		Defensive vs Aggressive	1.625	0.453	0.000*	5.081
	150 to 200	72	Defensive vs Turner	2.800	0.496	0.000*	16.446		Aggressive vs Turner	2.728	0.677	0.000*	15.295
			Aggressive vs Turner	1.561	0.522	0.003*	4.764	72	Defensive vs Turner	2.670	0.458	0.000*	14.439
			Defensive vs Aggressive	1.239	0.488	0.011*	3.452		Defensive vs Aggressive	−0.058	0.547	0.916	0.944
	Less 150	288	Defensive vs Turner	1.667	0.252	0.000*	5.295		Defensive vs Turner	1.784	0.303	0.000*	5.955
			Defensive vs Aggressive	0.966	0.277	0.000*	2.627	287^a^	Aggressive vs Turner	1.159	0.415	0.005*	3.185
			Aggressive vs Turner	0.701	0.269	0.009*	2.016		Defensive vs Aggressive	0.626	0.265	0.018*	1.869
Village^b^	Straight	72	Defensive vs Turner	1.859	0.459	0.000*	6.415		Aggressive vs Turner	0.958	0.410	0.020*	2.607
			Defensive vs Aggressive	1.342	0.441	0.002*	3.828	72	Defensive vs Turner	0.083	0.5525	0.880	1.087
			Aggressive vs Turner	0.516	0.427	0.227	1.676		Defensive vs Aggressive	−0.875	0.508	0.085	0.417
	300 to 800	72	Defensive vs Aggressive	2.934	0.767	0.000*	18.796		Defensive vs Aggressive	3.667	0.542	0.000*	39.121
			Defensive vs Turner	2.480	0.425	0.000*	11.936	72	Defensive vs Turner	1.917	0.513	0.000*	6.798
			Aggressive vs Turner	−0.454	0.554	0.412	0.635		Aggressive vs Turner	−1.75	0.800	0.029*	0.174
	200 to 300	71^a^	Defensive vs Turner	3.591	0.514	0.000*	36.255		Defensive vs Turner	3.917	0.692	0.000*	50.233
			Aggressive vs Turner	2.159	0.529	0.000*	8.662	72	Aggressive vs Turner	2.75	0.660	0.000*	15.643
			Defensive vs Aggressive	1.432	0.552	0.009*	4.186		Defensive vs Aggressive	1.167	0.631	0.064	3.211
	150 to 200	144	Defensive vs Turner	2.191	0.380	0.000*	8.948		Defensive vs Turner	1.396	0.410	0.001*	4.038
			Defensive vs Aggressive	1.649	0.440	0.000*	5.204	144	Defensive vs Aggressive	1.021	0.377	0.007*	2.776
			Aggressive vs Turner	0.542	0.372	0.145	1.719		Aggressive vs Turner	0.375	0.537	0.485	1.455
	Less 150	144	Defensive vs Turner	2.962	0.431	0.000*	19.336		Defensive vs Turner	3.042	0.531	0.000*	20.940
			Defensive vs Aggressive	2.943	0.529	0.000*	18.974	144	Defensive vs Aggressive	2.438	0.726	0.001*	11.444
			Aggressive vs Turner	0.238	0.303	0.432	1.268		Aggressive vs Turner	0.604	0.790	0.444	1.830

*Note.* * *p* < .05. Orders of comparison are based on odds ratios.

^a^1 observation was missing.

^b^GEE with the linear link function was used for naturalness (see Appendix B).

In the Village sections, where the controllers negotiated the road at a lower speed, the observed pattern with curvature outlined above, was not as apparent. This may be because all controllers negotiated the curves at a relatively low speed, thus reducing the effect of Curve Radius. Overall, the Defensive controller remained the most comfortable and natural, compared to the Aggressive and Turner controllers, indicated by the odds ratios for all Village sections. In contrast, not much difference was seen in the evaluation for comfort and naturalness between the Aggressive and Turner controllers, for the Village sections.

### The Influence of Personality Traits on Subjective Evaluation

Following data collection, participants were divided into two sub-samples, based on their average scores to the 20 AISS items. Evaluation of the controllers by the two sub-samples, providing the lowest (mean = 48.54, *N* = 13), and highest sensation seeking score (mean = 59.45, *N* = 11), was then assessed.

**Figure 9.**
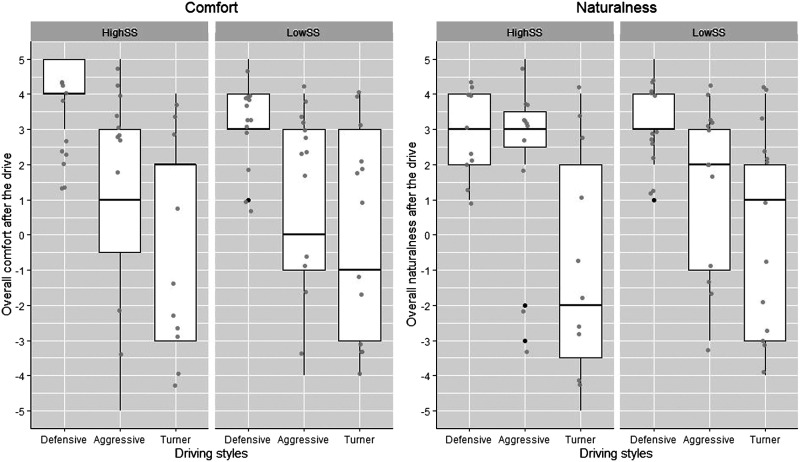
Overall comfort (left) and naturalness (right) evaluation of the driving styles from different sensation seekers.

Table 6 and Figure 9 show that the Defensive driving style was regarded as the most comfortable, for both the high and low sensation seekers. Interesting results were observed regarding the evaluation of naturalness. Low sensation seekers evaluated the Defensive as much more natural than the other two controllers, whereas high sensation seekers rated the Aggressive and Defensive driving styles about the same, in terms of naturalness. This finding also explains the bimodal pattern of evaluations on naturalness, shown in Figure 5 (right).

**TABLE 6:** The GEE Model Parameter Estimates and Odds Ratios Regarding Comfort and Naturalness for Low and High Sensation Seekers

	*N*	Driving Style	Coefficient	SE	Wald	Sig	Odds Ratio (OR)
Comfort							
Low sensation seekers	934^a^	Defensive vs Turner	1.809	0.346	27.305	0.000*	6.102
		Defensive vs Aggressive	1.356	0.378	12.875	0.000*	3.881
		Aggressive vs Turner	0.452	0.436	1.075	0.300	1.572
High sensation seekers	791^a^	Defensive vs Turner	2.153	0.296	53.039	0.000*	8.607
		Defensive vs Aggressive	1.507	0.281	28.865	0.000*	4.515
		Aggressive vs Turner	0.645	0.348	3.438	0.064	1.906
Naturalness							
Low sensation seekers	936	Defensive vs Turner	1.593	0.262	37.061	0.000*	4.919
		Defensive vs Aggressive	0.949	0.368	6.666	0.010*	2.583
		Aggressive vs Turner	0.644	0.463	1.933	0.164	1.904
High sensation seekers	789^a^	Defensive vs Turner	1.652	0.379	18.98	0.000*	5.217
		Aggressive vs Turner	1.258	0.370	11.584	0.001*	3.518
		Defensive vs Aggressive	0.394	0.334	1.390	0.238	1.483

*Note*. * *p* < .05.

^a^observations were missing, with the number of 2, 1, and 3, respectively.

## DISCUSSION

This driving simulator study examined users’ subjective evaluation of the driving style of three AV controllers, in terms of comfort and naturalness, when negotiating a range of rural and village sections of a UK road. The link between participants’ sensation seeking scores and their evaluation of these controllers was also investigated.

In terms of human- vs machine-like controllers, results showed that users preferred the two human-like AV controllers, in terms of both comfort and naturalness. Contrasting our findings with similar studies is challenging, as, at the time of writing, there are very few studies which have explicitly compared participant preferences for human-like and machine-like automated vehicle controllers. One exception is a study by Oliveira et al. (2019), who measured users’ trust towards a highly automated pod, which showed either human- or machine-like driving behaviours, when crossing a T-junction. In this study, human-like behaviour was produced by demonstrating a cautious ‘peeking’ behaviour by the pod, before it crossed the junction, while machine-like behaviour was produced by an assertive crossing, as if the road conditions were known to the automated pod. Oliveira et al. (2019) showed no difference in trust ratings for the two behaviours of the pod. There are two reasons why our study results are in contrast to those of Oliveira et al. (2019). One may be due to a difference in the concept used between our two studies: trust versus comfort and naturalness. The other may be because of the lower operating speed of the AV used in an urban road, by Oliveira et al, compared to the higher travelling speeds of our vehicle, travelling in rural road sections. This contrast in results illustrates the importance of considering the scenarios used to evaluate AV-driving styles in such studies, since they vary across different road environments, based on both geometry and posted speed limit, which clearly influences any subjective assessment and evaluation (Hajiseyedjavadi et al., 2021). Further work on the influence of different scenarios on subjective appraisals of human- vs machine-like AV-driving styles, should clarify this.

Overall, participants rated the Defensive controller more comfortable than the other two controllers, while both the Defensive and Aggressive controllers were assessed as more natural than the Turner. This suggests that there may be a distinction between what human evaluators consider a comfortable versus natural driving style, which is perhaps in contrast to the suggestion made by Elbanhawi et al. (2015), who regarded natural, or familiar, driving manoeuvres as one contributor to driving comfort. Our results suggest that comfort and naturalness of a controller should not be used interchangeably in such research, and that while human-like driving styles can be considered as equally more natural than a ML-based controller, they are not necessarily as equally comfortable. Therefore, factors which contribute to the comfort of a controller are not the same as those that determine its naturalness.

Regarding how road geometries and vehicle kinematics affected subjective ratings, our results show that variations in speed potentially had a greater influence on evaluation of comfort and naturalness of the controllers, when compared to differences in lateral offset. This was especially the case for the rural sections, which contained roads of tighter curvature, and higher speed. These results are in line with the work of Hajiseyedjavadi et al. (2021), who found that their model-based human-like AV-driving controllers were assessed as less pleasant when negotiating narrower curves. These authors also found that a more rigid controller, which always followed the centre of the lane, received better evaluations. Together, these results suggest the influence of vehicle kinematics and road geometry on subjective evaluation of AV controllers. Moreover, our results showed little difference in lateral kinematic features of the three controllers, which was also reflected in the evaluations. Therefore, future studies need to examine the effect of more pronounced lateral offset on subjective evaluation, especially since maintaining sufficient and safe distance to road edges is thought to enhance driving comfort (Summala, 2007).

We found an interesting interaction between personality trait and evaluation of the controllers, with high sensation seekers rating the Aggressive driving style (which was a recording of another representative high sensation seeker) as natural, which was not the case for low sensation seekers. As naturalness in this study was defined as a driving style that is ‘*closest to your own driving’,* it is interesting to see this strong influence of personality traits on driving style and preference. The distinction between comfort and naturalness as concepts is also highlighted here because there was no difference in the two groups, when evaluating the comfort of the Aggressive driving style. In other words, while the high sensation seekers thought the Aggressive driving style was natural, they did not find it comfortable. These results highlight the value of personalisation of automated controllers, to benefit the range of preferences by consumers with varying personality traits, notwithstanding their safety considerations.

### Limitations

One limitation of the present study is the motion-planning performance of the Turner controller, which was developed using a small number of participants. Moreover, although the motion planner’s output consists of a series of aim speeds and positions, we only used simple controllers that were manually calibrated for the automated vehicle to drive, using this data flow. Thus, a future study could use more data to train the motion planner and consider a better approach for implementing the controllers.

As with all controlled driving simulator studies, there are caveats regarding the relevance and generalisability of these findings, and their implications with respect to real-world AV controllers. Creating very realistic controllers was possible in this driving simulator study, due to its advanced motion-controller capabilities. However, future studies would benefit from evaluating these sorts of controllers in more real-world settings, also assessing how such evaluation is affected by other real-world factors, such as different road surfaces, or presence of other roadside objects and road users.

## Conclusions

Participants rated the two human-like driving styles as more natural, compared with the less human-like, ML-based, controller. Most participants also rated the Defensive driving style (gentler speed profiles) as more comfortable than the Aggressive controller (higher accelerations and more sudden braking profiles). This study shows, for the first time, that participants are able to distinguish between the natural driving manoeuvres of humans and the more machine-like negotiations of an artificial controller. In addition, we illustrate that there is a more complex relationship between concepts such as comfort and naturalness when evaluating automated vehicle controllers.

## Figures and Tables

**Figure 10.**
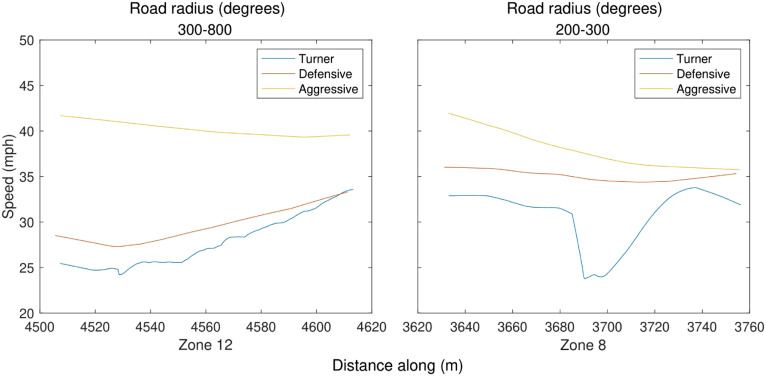
Vehicle speed of three controllers in the two village road curves.

## APPENDIX A: Cluster Analysis for Categorising Driving Behaviour

From a previous study of the project, driving behaviours were collected from a sharp curve (radius < 150 m), a zone with parked cars (length = 162.68 m), and the entire drive. The variables used as behavioural indicators for clustering were: root mean square of speed, standard deviation of longitudinal acceleration, and standard deviation of yaw rate. The k-means clustering analysis was conducted.

## APPENDIX B: Configuration of the GEE Model

The working correlation matrix was specified as *exchangeable*, which characterises the correlation structure of multiple observations within a participant as the same. As the comfort and naturalness were rated using an ordinal Eleven-point Likert scale, the distribution of the dependent variable was specified as a *multinomial* distribution. A link function is used to characterise the relationship between the mean of the response (i.e., subjective ratings) and the linear predictor (i.e., controllers). The *ordered logit regression* was specified as the link function.

However, in some Village road segments, including the Straight road sections, the 200–300 Radius sections, and the 150–200 Radius sections, the statistical model did not provide valid results, because the participants’ responses to the three controllers in these sections showed very similar patterns, which resulted in collinearity. For example, in the Curve Radius 150–200 section, most responses clustered between 2 to 5 for all three driving styles. Therefore, we treated the data as continuous in these road sections, to allow statistical comparisons. The distribution was specified as normal in the GEE.

## APPENDIX C: Vehicle Kinematics Inspection for the Two Village Road Sections with Unexpected Assessments

Inspection of the vehicle-based metrics for these two particular road sections (Figure 10) showed that the Aggressive controller’s speed was markedly higher than that of the two other controllers in the 300–800 Radius section, and higher than the designated speed limit of 30 mph. A sudden fluctuation of speed for the Turner controller was also seen in the 200–300 Radius section, although it was within the speed limit of 40 mph. Further inspection of the simulated scene did not show the presence of any unusually placed road furniture, such as parked cars. A possible explanation here is that the Turner controller did not look far enough ahead to smooth out the speed changes, and was also inadequately sensitive to roadside furniture. Regardless, these results show that the effect of these kinematic changes were clearly felt by our users, which can possibly explain their evaluation of the controllers for these two sections.
